# Using a serious game for a brief assessment of dark personality in the workplace

**DOI:** 10.3389/fpsyg.2025.1686784

**Published:** 2026-01-26

**Authors:** María Patricia Navas, Elena Fernández-del-Río, Pedro J. Ramos-Villagrasa

**Affiliations:** Facultad de Ciencias Sociales y del Trabajo, Departamento de Psicología y Sociología, Universidad de Zaragoza, Zaragoza, Spain

**Keywords:** dark personality, gamification, game-related assessment, gamified assessment, job performance, counterproductive work behaviors

## Abstract

**Introduction:**

The assessment of dark personality using self-report questionnaires suffers limitations due to social desirability, bias, and response faking, particularly in organizational contexts. This research examines the psychometric properties of an extended version of VASSIP, a gamified assessment to briefly measure dark personality through an immersive situational judgment test (SJT).

**Methods:**

A sample of 395 Spanish workers (47.4% female, *M*_job experience_ = 10.4 years) participated in the study, completing questionnaires of the target variables and playing the gamified assessment.

**Results:**

The hard-gamified extension of VASSIP has a unidimensional factor structure with 5 items. Validity was supported by direct associations with Honesty-Humility, moral disengagement, task performance, and counterproductive work behaviors (CWBs).

**Discussion:**

Therefore, this measure appears to be valuable for briefly assessing dark personality, although its predictive capacity could be optimized. Its situational approach offers a more nuanced understanding of how individuals manifest dark personality in workplace scenarios.

## 1 Introduction

The prediction of job performance is a key issue in the study of work behavior. In that sense, personality is one of the main predictors of performance across most occupations and settings (Salgado and Moscoso, 2019). Specifically, meta-analytic reviews have consistently confirmed the validity of the ‘Big Five’ personality traits for predicting performance, highlighting conscientiousness and neuroticism as the best correlates (Judge et al., 2013). Recent research has turned its attention to dark personality, a cluster of subclinical, socially undesirable traits associated with various antisocial behaviors that can impact organizational outcomes (Szabó et al., 2023). By considering dark personality in addition to the Big Five, researchers are finding an increase in the explained variance of job performance (Fernández-del-Río et al., 2020). However, the frequent use of self-reported questionnaires in organizational settings limits the assessment of these traits. Respondents tend to misrepresent their responses to provide a better view of themselves (Wille et al., 2023), so we need alternative measures that prevent fake scores (Miller et al., 2019; Walker et al., 2022). One of these alternatives may be game-related assessments or GRAs (Ramos-Villagrasa and Fernández-del-Río, 2023). The study reported here analyses the performance of a GRA for the brief assessment of dark personality in organizational contexts.

### 1.1 Dark personality

Most research on dark personality in the work context has been based on the Dark Triad (Paulhus and Williams, 2002). The Dark Triad, focused on self-interest, describes a personality profile that shares the subclinical characteristics of three personality traits: Machiavellianism, psychopathy, and narcissism. Individuals with these traits tend to be insensitive, selfish, and malevolent in their interpersonal relationships. Recently, there has been a scholarly consensus to consider everyday sadism as part of this construct, renamed Dark Tetrad (Paulhus, 2014). Along these lines, several papers have recently advocated the existence of a latent (super) malevolence factor or core of the dark personality called the ‘D factor’, similar to the proposal of a General Personality Factor (e.g., van der Linden et al., 2010). Different dark traits (i.e., psychopathy, Machiavellianism, narcissism, sadism) would emerge from this factor as specific manifestations (Moshagen et al., 2020). Therefore, according to these authors, assessing dark personality by an omnibus score could contribute to screening for the shared malevolence that underlies different dark personality profiles. As an alternative to this dark personality approach, some authors argue that low scores on the Honesty-Humility (H-H) factor of the HEXACO personality model (Lee and Ashton, 2014) or low scores on the Big Five Agreeableness factor (Vize et al., 2021) offer a better explanation for the common core of malevolence considered in the D factor. Despite the high percentage of shared variance, Horsten et al. (2024, p. 400) argue that the lower pole of H-H and Agreeableness “do not represent D.”

### 1.2 Individual differences in personality and job performance

Job performance is considered the ultimate criterion in human resource management (Organ and Paine, 1999). Empirical evidence about the relationship between dark and light personality and job performance is far from conclusive (Moscoso and Salgado, 2004; Fernández-del-Río et al., 2020; Zettler and Solga, 2013). One possible explanation for the differences identified is the consideration of the multidimensional nature of performance. Thus, although job performance comprises behaviors of workers that contribute to organizational goals (Campbell and Wiernik, 2015), three main domains stand out (Sackett and Lievens, 2008): task performance, contextual performance, and counterproductive work behaviors. Task performance (TP) refers to behaviors that support the “technical core” of the organization, involving the execution and maintenance of processes, formally recognized as job requirements (Borman, 2006; Motowidlo et al., 2014). Contextual performance (CP; Smith et al., 1983) is behavior that contributes to organizational goals by collaborating socially and psychologically through initiative and cooperation (Koopmans et al., 2011). Counterproductive work behaviors (CWBs) are employees’ intentional actions that harm the organization and/or its members (Sackett and DeVore, 2001). These behaviors are divided into deviations directed at individuals—CWBI—or the organization—CWBO— (Bennett and Robinson, 2000).

Research on the relationship between TP and dark personality has shown mixed results. O’Boyle et al. (2012) conducted a meta-analysis that found small but significant effects of Machiavellianism (*r* = −0.06) and psychopathy (*r* = −0.08) on TP, while narcissism was not significant (*r* = −0.02). However, a recent primary study developed in Spain by Fernández-del-Río et al. (2020) found that narcissism (*β* = 0.23) and Machiavellianism (*β* = 0.10) positively predicted TP, whereas psychopathy (*β* = −0.14) and sadism (*β* = −0.11) were negative predictors. The inconsistency of the results suggests the need for further research in this field.

Evidence also suggests a negative relationship between dark personality and CP. Judge et al. (2006) found that narcissism affected this performance more negatively than TP. Becker and O'Hair (2007) found that Machiavellianism negatively affected civic behaviors, especially toward the organization. Fernández-del-Río et al. (2020) confirmed this relationship (*β* = −0.18) but found narcissism to be a positive predictor (*β* = 0.34), possibly due to the characteristics of the narcissistic pattern (i.e., high sense of self-importance, conceit, ostentation, etc.).

The scientific literature has paid increasing attention in recent years to the relationship between dark personality and CWBs, which are widespread in the workplace and pose a serious threat to both organizational performance and employee well-being (Duradoni et al., 2023). According to a meta-analysis by O’Boyle et al. (2012), the three components of the Dark Triad are positively, albeit weakly, related to this type of negative behavior. More recently, in the first study that considered sadism as a predictor of job performance, Fernández-del-Río et al. (2020) found that precisely this dark personality pattern best explained CWBs. However, we wonder whether some variables, such as moral disengagement, could influence the relationship between dark personality and undesirable behaviors in the workplace. Moral disengagement—a cognitive process by which people distance themselves from their internal moral standards to behave unethically (Navas et al., 2024)—may increase the explained variance of immoral behaviors in dark personalities in the workplace, as in other contexts (e.g., Egan et al., 2015). In any event, we need cumulative evidence of the relevance of moral disengagement in predicting workplace deviance (Ramos-Villagrasa et al., 2025).

### 1.3 Measuring dark personality in the workplace: traditional instruments and new alternatives

In the work setting, most research on dark personality has been developed using self-report measures, although they differ in length, reliability, and validity. Researchers, namely Uppal (2022), Ramos-Villagrasa et al. (2025), and Sekhar and Uppal (2024), have tended to use general scales, rather than work-specific ones—such as the Dirty Dozen (DD; Jonason and Webster, 2010), the Short Dark Tetrad (SD4; Paulhus et al., 2021), and Dark Core Scale (DCS; Moshagen et al., 2020). A notable exception is the Dark Tetrad at Work Scale (DTW; Thibault and Kelloway, 2020), whose use is increasing (e.g., Fernández-del-Río et al., 2020; Longpré and Turner, 2024). Although long questionnaires are preferable when there are no time constraints, extensive tests are not always necessary in the organizational domain, and brief scales are a significant time-saver (DeNisi and Murphy, 2017). There is a call for shorter personality measures (Rammstedt and Beierlein, 2014), driven in part by the recognition that alleged limitations, including lower reliability or criterion correlations, are frequently misunderstandings (Ziegler et al., 2014).

A common challenge in personality measurement within organizational contexts is that applicants tend to present themselves in a positive light (Birkeland et al., 2006). However, this concern is amplified when assessing dark personality, as the traits themselves are socially undesirable. Given that personality assessments are leveraged for critical personnel decisions (e.g., selection, promotion, development) in the workplace, minimizing such impression management is crucial, particularly when attempting to identify these subtle yet impactful characteristics. Thus, reliable and valid alternative measures are considered necessary in contexts with consequences for the person being assessed (Miller et al., 2019; Walker et al., 2022). GRAs could be an alternative technique that acts as a “shadow assessment system” to obtain more comprehensive and accurate information from candidates by reducing social desirability and response faking (Landers and Collmus, 2022; Melchers and Basch, 2022).

According to the main theoretical models on deception (Ellingson and McFarland, 2011; Levashina and Campion, 2006; Tett and Simonet, 2011), this behavior is determined by three interrelated factors: the capability, motivation, and opportunity to deceive. Capability refers to the individual’s cognitive ability to identify which responses will be perceived as most socially desirable; motivation depends on both personal characteristics (e.g., competitive orientation or personality traits) and situational factors (e.g., job importance or organizational attractiveness); and opportunity relates to the extent to which the assessment format allows responses to be adjusted to create a favorable impression (Ohlms et al., 2024). In this context, situational judgment tests (SJTs) represent a significant advance over traditional personality questionnaires. While self-reports are based on introspective judgments that are easily influenced by social desirability, SJTs place the individual in specific scenarios that require selecting or evaluating hypothetical behaviors, which reduces the transparency of the evaluation criteria and, therefore, the opportunity to simulate (Lievens and Motowidlo, 2016; Mussel et al., 2016). Furthermore, by involving the interpretation of contexts and the application of knowledge about which behaviors are effective, SJTs tend to be less vulnerable to falsification and offer greater ecological validity, as they approximate the way people make decisions in social or work situations (Olaru et al., 2019).

On this basis, gamified SJTs take this approach a step further by introducing interactive and dynamic environments that significantly modify the three components of cheating. First, the high cognitive load and complexity of the environment reduce the ability to identify which response will be most highly valued (Altomari et al., 2023). Second, playful immersion decreases the motivation to falsify, as participants focus on performance within the game rather than managing their self-image (Bhatia and Ryan, 2018). This reduced vulnerability to simulation is particularly relevant in the assessment of dark personality, as it is associated with greater motivation and the ability to manipulate self-presentation in selection contexts (Roulin and Krings, 2016). In these contexts, where social desirability is high, gamified SJTs offer a format in which strategic manipulation is more difficult and in which the decisions and behaviors observed more genuinely reflect the characteristics of dark personality. Finally, the opportunity is limited by the low transparency of the evaluation criteria, which are integrated into the narrative or dynamics of the game and are not evident (Woods et al., 2020). Taken together, these factors favor more spontaneous and authentic responses than those generated in classic personality questionnaires. Therefore, the integration of the fundamentals of falsification theory and situational assessment allows us to argue that gamified SJTs are a particularly relevant tool for the valid and ethical assessment of dark personality in organizational contexts.

Gamified SJTs belong to GRAs, a set of assessment methods that utilize games or gamified tools (Ramos-Villagrasa et al., 2022). They can be classified on a continuum according to their degree of playfulness (Ramos-Villagrasa and Naryniecki, 2025). Within this continuum, tests that incorporate game-like elements in their assessment are referred to as serious games and can, therefore, be considered psychological assessment methods in their own right, with adequate predictive validity (Harman and Brown, 2022; Hilliard et al., 2022). There are three types of serious games: (1) *soft gamified assessments*, which are similar to traditional assessments but include gamification elements like music, stories, or score points; (2) *hard gamified assessments*, where gamification is fundamental to the assessment design; and (3) *game-based assessments*, which are structured as actual games. Therefore, a gamified SJT is classified into one of these types depending on its degree of playfulness. Compared to traditional assessment methods, such as questionnaires, serious games enhance individuals’ reactions (Ellison et al., 2020), mitigate bias (Landers and Collmus, 2022), and reduce faking behavior (Melchers and Basch, 2022; Ohlms et al., 2025).

Research on GRAs for assessing personality is scarce and always focused on “bright” personality (Barends and Ohlms, 2025). The closest example to date is *Building Docks*, a hard gamified assessment designed to measure H-H (Barends et al., 2022). Although dark personality overlaps with H-H, the latter appears to be functionally and nomologically distinct, and dark personality outperformed in the prediction of aversive behaviors, like CWBs (Horsten et al., 2021). Therefore, there is still room to evaluate dark personality through gamification. In this paper, we present an extension of *VASSIP* (Ramos-Villagrasa et al., 2024), a soft-gamified assessment that evaluates the Big Five model, expanding its scope to measure dark personality.

### 1.4 The present study

Addressing the need for alternative dark personality assessment measures and leveraging the advantages of GRAs, this research aims to evaluate the performance of a hard-gamified assessment designed for the brief appraisal of dark personality in organizational contexts. To achieve this, we will develop a dark personality measure, integrate it into an existing gamified assessment, and assess its functioning.

The development of the dark personality measure is based on situational judgment test (SJT)-type items (Lievens et al., 2008). SJTs, which present participants with “short domain-relevant situational descriptions and various response options to deal with the situations” (Herde et al., 2019, p. 66), have great potential for assessing personality (Lievens et al., 2021). The advantage of SJT-type items is that evaluees tend to rate them better, although they are often less reliable (internal consistency) than other scales due to the methodology used to design them (Kasten and Freund, 2016). Furthermore, McDaniel et al.’s (2007) meta-analysis found that SJT-type items had a mean observed correlation of 0.20 for predicting job performance and high face and content validity, so incorporating these types of items could be advantageous for the gamified assessment of dark personality in the work environment. This is especially relevant in contexts such as recruitment, where the use of gamified tests can improve validity, diversity, and candidate reactions (Van Iddekinge et al., 2023).

The process of gamifying the SJT items for inclusion in VASSIP was based on storyfication (Ohlms et al., 2024), which means incorporating them into the narrative so that the questions posed to the individuals being assessed become part of the story without substantially altering its development. The integration of the gamified SJT for measuring dark personality in the serious game VASSIP transforms it into a hard-gamified assessment (Ramos-Villagrasa and Naryniecki, 2025). This is because the described situations are embedded within the assessment’s narrative, such that the questions can only be answered by considering the gamified elements. An additional advantage is that this approach allows for the assessment of both the Big Five and dark personality using a single instrument.

To examine the functioning of the hard-gamified assessment, we will evaluate dimensionality, construct validity, and criterion validity. Regarding dimensionality, given the aim of creating a brief instrument, we intend to develop a unidimensional, general measure of dark personality for initial assessment. A more precise measure would require greater length, and we must first demonstrate that GRAs offer advantages in assessing this type of personality. For construct validity, we will test its convergence and divergence with a self-report measure of dark personality and measures of “bright” personality. Finally, we will examine criterion validity by analyzing whether the dark personality measure plays a role in the prediction of the different dimensions of job performance (i.e., TP, CP, and CWBs).

## 2 Method

### 2.1 Sample

To test the factor structure and construct validity of the VASSIP measure of dark personality, an a priory power analysis was conducted using GPower. The significance level (*α*) was set at 0.05, and the desired statistical power (1 – *β*) was 0.80. Based on an expected effect size of 0.30, the power analysis indicated a required sample size of *N* = 347. Thus, 410 workers living in Spain and fluent in Spanish were recruited through the Prolific research platform. Prolific ensures a controlled online environment with strict participant verification and in-study quality checks, providing high-quality data. Participants were informed about the study’s purpose and their rights according to APA ethical standards before deciding to participate. After eliminating participants with more than 5% of missing responses and those who failed the attentional task, the final sample comprised 395 workers (47.4% female, *M*_age_ = 34.2 years, *SD*_age_ = 10.4, *M*_job experience_ = 10.83 years, *SD*_job experience_ = 9.5).

### 2.2 Measures

#### 2.2.1 Attention check

The questionnaire with the target variables included a question stating, “Please select Disagree as the answer to this question.” Fifteen participants failed the check and were removed from the study.

#### 2.2.2 Sociodemographic

Participants were asked about their gender, age, level of education, job experience, and current job position.

#### 2.2.3 Gamified assessment of personality

As previously described, the gamified personality measure is an extension of VASSIP (Ramos-Villagrasa et al., 2024), a soft-gamified assessment that evaluates “bright” personality using the Big Five model. The Big Five measure retains its original format, with items and response format identical to the short version of the BFI-2 (Soto and John, 2017). For dark personality, the authors of this paper developed a gamified SJT.

The gamified elements of the original version by Ramos-Villagrasa et al. (2024) are still present in this one. The first version, storyfication, consists of embedding the assessment into a science fiction story, in which the person being assessed is hired to work in a space base whose security is compromised. During the game, the person being tested must make decisions until reaching one of the three outcomes of the story (see Ohlms et al., 2024, for more information about storified assessments). Immersion in the test is achieved through images and music that are not part of the assessment but make it easier for the evaluee to feel that they are part of a story and not part of an assessment process. Finally, VASSIP incorporates decision-making and some simple games that are not part of the assessment but help the participants perceive the experience more as a game than a personality assessment. As developing the measure of dark personality is among the objectives of the present study, information about the measure is described in detail in the following sections. Given the changes performed on the original VASSIP scale, we consider the present version a hard-gamified assessment because the measurement is embedded in the game narrative (Ramos-Villagrasa and Naryniecki, 2025).

#### 2.2.4 Honesty-Humility (H/H)

This dimension of the HEXACO model was measured with the Spanish version of the corresponding subscale of HEXACO-100 (Lee and Ashton, 2004; Spanish version by Romero et al., 2015). The internal consistency of this scale was *ω* = 0.76 (McDonald’s omega) and *α* = 0.74 (Cronbach’s alpha). A sample item is “If I want something from a person I dislike, I will act very nicely toward that person to get it.”

#### 2.2.5 Moral disengagement

We used the Spanish version of the 8-item Propensity to Moral Disengagement Scale (Moore et al., 2012; Spanish version by Navas et al., 2024). It is rated on a 7-point Likert scale ranging from 1 (*strongly disagree*) to 7 (*strongly agree*). The internal consistency of this scale was *ω* = 0.70 (McDonald’s omega) and *α* = 0.67(Cronbach’s alpha). A sample item is “It is okay to spread rumors to defend those you care about.”

#### 2.2.6 Self-reported measure of dark personality

We applied the Spanish version of the Short Dark Tetrad (SD4; Paulhus et al., 2021) used by Ramos-Villagrasa et al. (2025). This scale comprises 28 items rated on a 5-point Likert-type scale, ranging from 1 (*strongly disagree*) to 5 (*strongly agree*). The internal consistency of this scale was *ω* = 0.72 for Machiavellianism, 0.74 for narcissism, 0.77 for psychopathy, and 0.78 for sadism (McDonald’s omega); and *α* = 0.72 for Machiavellianism, 0.73 for narcissism, 0.76 for psychopathy, and 0.77 for sadism (Cronbach’s alpha). A sample item is “Watching a fistfight excites me.”

#### 2.2.7 Big Five

As the present version of VASSIP is based on the original one developed by Ramos-Villagrasa et al. (2024), it includes a gamified version of the short version (30 items) of the BFI-2-S (Soto and John, 2017) based on storyfication, immersion, and the inclusion of game dynamics that are not part of the assessment, while the items and response format are identical to the original test. The authors of VASSIP reported similar results to those of the original scale, with a slightly higher mean score in Conscientiousness. Responses are rated on a 5-point Likert-type scale, ranging from 1 (*strongly disagree*) to 5 (*strongly agree*). The internal consistency of this scale was ω = 0.82 for Negative Emotionality, 0.73 for Extraversion, 0.80 for Open-Mindedness, 0.69 for Agreeableness, and 0.81 for Conscientiousness (McDonald’s omega), and α = 0.82 for Negative Emotionality, 0.72 for Extraversion, 0.79 for Open-Mindedness, 0.68 for Agreeableness, and 0.81 for Conscientiousness (Cronbach’s alpha). A sample item is “Is full of energy.”

#### 2.2.8 Task performance and contextual performance

We applied the Spanish version of Individual Work Perfomance Questionnaire (IWPQ), developed by Koopmans (2015) and translated into Spainish by Ramos-Villagrasa et al. (2019). The IWPQ comprises two subscales: Task Performance (5 items, ω = 0.87 and α = 0.86). and Contextual Performance (13 items, ω = 0.86 and α = 0.86). Participants answered on a 5-point scale ranging from 0 (*never*) to 4 (*often*). A sample item of Task Performance is “I managed to plan my work so that I finished it on time.” A sample of Contextual Performance item is “I took on challenging tasks when they were available.”

#### 2.2.9 Counterproductive work behaviors

We applied the Workplace Deviance Scale (WDS; Bennett and Robinson, 2000; Spanish version by Fernández del Río et al., 2021). The WDS comprises two subscales: Organizational Behaviors or CWBO (12 items, ω = 0.82 and α = 0.80) and Interpersonal Behaviors or CWBI (7 items, ω = 0.89 and α = 0.88). Participants answered on a 7-point scale ranging from 1 (*never*) to 7 (*daily*). A sample item is “Have taken property from work without permission.”

### 2.3 Procedure

Participants were randomly divided into two groups: (1) Condition 1 accounted for 52.7% of the sample, who first completed an online questionnaire related to the target variables and subsequently played the gamified assessment; and (2) Condition 2, which comprised the remaining 47.3% of the participants, who first played the gamified assessment and then completed the online questionnaire. This approach, which is a standard method for counterbalancing two tasks in empirical designs, ensures that any potential influence of task order is distributed across participants rather than confounding the results (Kirk, 2013).

This research was approved by the ethics committee of Aragón (CEICA, ref. PI24/123).

### 2.4 Analysis

#### 2.4.1 Item generation and content validation

The formal procedure proposed by Haynes et al. (1995) was carried out to provide apparent validity to the content of the developed SJT. For this purpose, a common empirical methodology for developing SJTs, known as hit-rate analysis, was used (Moore and Benbasat, 1991).

Fifteen situational scenarios requiring decision-making were designed, each one with three response options. These options represented a low, medium, and high score in dark personality. After an internal review, scenarios were sent to four external experts (three academics and one HR professional) who assessed the value they would give to each response alternative (low, medium, or high). After analyzing the responses, the experts and researchers concluded that in the gamified assessment, a total of 12 items reached acceptable levels of congruence during the content validation procedure (Kendall’s *W* = 0.63 for the gradation of the intensity of the responses) in the SJT format according to Cicchetti (1994) guidelines.

#### 2.4.2 Factor structure and validity of the gamified assessment

A confirmatory factor analysis (CFA) was conducted to assess the adequacy of the structure of the gamified SJT with the data provided by the participants. Diagonally Weighted Least Squares (DLWS) estimates and robust statistics were used to address non-normality of the data and fit indices, as recommended by Hu and Hu and Bentler (1999). More specifically, the following criteria were considered for optimal fit: χ^2^/*df* < 2–3, CFI > 0.95, RMSEA < 0.06, SRMR < 0.05; and for acceptable or reasonable fit: χ^2^/*df* < 4, CFI > 0.90, RMSEA < 0.08, SRMR < 0.08 (Byrne, 2012).

To establish the construct validity of the new hard-gamified extension of VASSIP, participants’ responses were used to perform descriptive analyses, mean comparisons, correlations, and linear and hierarchical regressions, with a set of well-established measures used as independent variables.

All analyses were performed using JAMOVI statistical software.

## 3 Results

### 3.1 Factor structure of the items that comprise the gamified assessment

The 12 items that constitute the gamified STJ were subjected to a CFA where each item loads on a first-order factor representing dark personality. The fit indices in the CFA for this model were not optimal, *χ*^2^ (80.8, 54) = 1.55, *p* < 0.05, CFI = 0.74, RMSEA = 0.03 [0.01, 0.05], SRMR = 0.08. Consequently, items whose estimators neither showed a significant contribution to the model (*p* > 0.05, *n* = 5 items = i1, i2, i4, i9, and i12) nor significant correlations with the dimensional scores obtained on the self-report assessing dark personality (SD4, *n* = 2 items = i3 and i6) were removed, so that the final items can be seen in Table 1.

**Table 1:** Correlations among the 12 items of the *VASSIP* measure of dark personality and the four dimensions of SD4.

Variables	1	2	3	4	5	6	7	8	9
1. SD4_Machiavellianism	--								
2. SD4_Narcissism	0.29***	--							
3. SD4_Psychopathy	0.27***	0.42***	--						
4. SD4_Sadism	0.45***	0.25***	0.42***	--					
5. Item 1	0.05	0.13**	−0.01	−0.01	--				
6. Item 2	0.02	−0.01	0.04	0.03	0.04	--			
7. Item 3	0.05	0.12*	0.02	0.02	0.08	−0.00	--		
8. Item 4	0.02	0.22***	0.09	0.04	0.04	−0.01	0.00	--	
9. Item 5	0.16*	0.20***	0.13*	0.23***	0.09	0.09	0.10*	0.13	--
10. Item 6	0.03	0.04	0.06	0.08	0.04	0.03	0.08	0.06	0.05
11. Item 7	0.15**	0.08	0.13*	0.09	0.11**	−0.02	0.04	0.07	0.07
12. Item 8	0.16**	0.07	0.08	0.14***	0.12*	0.08	−0.05	0.03	0.09
13. Item 9	−0.04	0.03	0.06	0.07	0.10*	−0.07	0.10*	0.08	0.02
14. Item 10	0.22***	0.18***	0.10*	0.35***	−0.05	−0.01	0.01	0.09	0.11*
15. Item 11	0.24***	0.04	0.07	0.20***	0.06	0.01	−0.04	−0.07	0.13**
16. Item 12	0.12	−0.02	0.04	0.16***	0.13**	−0.07	0.08	0.05	−0.00

*p < 0.05. **p < 0.01. ***p < 0.001.

**Figure 1:**
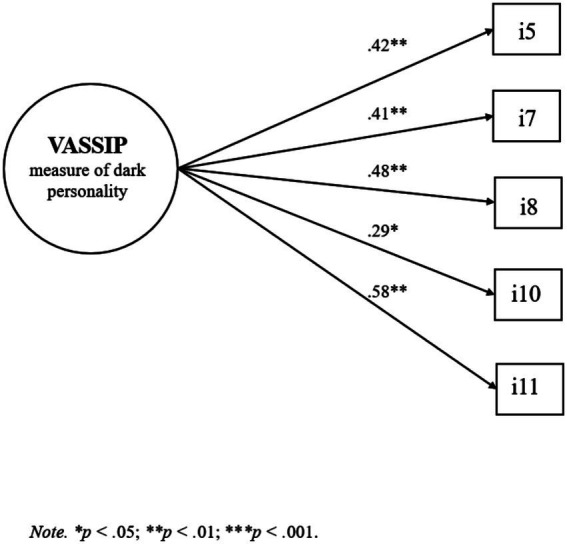
Confirmatory factor structure of the items comprising the gamified version of the VASSIP measure of dark personality.

The reduction from 12 to 5 items was guided by well-established psychometric criteria (DeVellis, 2017). Items that did not load significantly on the factor, nor correlate meaningfully with the dimensional scores obtained from the reference self-report, were excluded to improve construct validity and model fit. This approach also follows the principle of parsimony, aiming for a concise instrument that retains the core variance of the latent construct while minimizing redundancy (MacCallum et al., 1992). Subsequently, the five resulting items composing the gamified assessment of dark personality (i5, i7, i8, i10, and i11) were subjected to an CFA, showing optimal fit indices, *χ*^2^(17.3, 14) = 1.27 *p* > 0.05, CFI = 1.00, RMSEA = 0.00 [0.00, 0.02], SRMR = 0.02. The items are presented in the Supplementary material. The estimators of each item for the model are presented in Figure 1.

### 3.2 Descriptive statistics

The descriptive statistics (*M*, *SD*, skewness, kurtosis) presented in Table 2 indicate that, except for H-H (*W* = 0.99, *p* = 0.11), the data of the variables did not follow a normal distribution. Statistically significant differences between men and women were found in age, agreeableness, Machiavellianism, psychopathy, moral disengagement, and CWBI, albeit all with small effect sizes. In addition, negative emotionality (*d* = 0.24), dark personality—assessed using the gamified assessment (*d* = 0.32)—and sadism (*d* = 0.49), showed significant differences between men and women with a moderate effect size. Men displayed higher mean scores in dark personality, and women showed significantly higher mean scores in negative emotionality.

**Table 2:** Descriptive statistics and gender comparisons for all study variables.

Variables	*M*	*SD*	Skewness	Kurtosis	*Mann–Whitney U*	*p*	Cohen’s *d*
Gender	0.47	0.50	0.10	−2.00			
Age	35.23/33.08	10.83/9.84	0.92	0.09	16689	0.040	0.12
Dark personality (VASSIP)	9.47/7.77	3.11/2.45	0.85	0.80	12117	0.001	0.32
SD4_Machiavellianism	3.27/3.06	0.63/0.66	−0.35	0.35	15824	0.005	0.16
SD4_Narcisism	2.49/2.36	0.66/0.71	−0.04	−0.54	17303	0.135	0.08
SD4_Psychopathy	1.67/1.47	0.65/0.47	1.36	2.11	15588	0.002	0.17
SD4_Sadism	2.45/1.79	0.83/0.54	0.88	0.92	9584	0.001	0.49
Honesty-Humility	54.69/55.62	9.07/8.57	−0.17	0.00	17677	0.240	0.06
PMD	19.64/17.88	6.60/5.82	0.76	1.47	16329	0.022	0.13
Negative Emotionality	14.93/16.94	4.75/4.88	0.34	−0.57	13534	0.001	0.24
Extraversion	18.94/18.78	4.27/4.30	−0.10	−0.39	17290	0.621	0.02
Open-Mindedness	23.05/23.54	4.47/4.06	−0.56	−0.30	16864	0.378	0.05
Agreeableness	22.91/23.93	3.37/3.49	−0.41	−0.01	14735	0.004	0.17
Conscientiousness	21.19/21.32	4.58/4.60	−0.07	−0.57	17399	0.694	0.02
TP	3.12/3.22	0.63/0.68	−0.93	1.53	16872	0.058	0.11
CP	2.74/2.87	0.73/0.73	−0.62	0.33	17015	0.079	0.10
CWBO	25.83/25.43	10.27/8.79	0.86	0.55	17693	0.253	0.06
CWBI	10.54/8.84	5.72/4.28	3.57	17.10	15892	0.030	0.16

Gender: 0 = men; 1 = women, in columns with two values, the first one refers to men and the second one to women. PMD: propensity to moral disengagement; TP: Task Performance; CP: Contextual Performance; CWBO: counterproductive work behaviors targeting the organization; CWBI: counterproductive work behaviors targeting individuals.

The assessment of dark personality through the gamified assessment revealed significant differences according to application condition, where Condition 2 (playing the gamified assessment first, answering the self-report questionnaire afterwards) obtained higher scores on dark personality measured through the gamified assessment (Mann–Whitney *U* = 15121, *p* = 0.003, *d* = 0.17).

### 3.3 Correlations and regression analyses

Next, Spearman correlations were conducted to determine whether the scores on the gamified dark personality assessment were significantly associated with scores on Machiavellianism, narcissism, psychopathy, and sadism of the self-reported assessment and with the several dimensions of job performance. Spearman’s correlations (*ρ*) are reported together with their 95% confidence intervals obtained via bootstrapping (1000 samples), providing a more reliable estimation of the associations. The associations between the variables are presented in Table 3. The relationships between dark personality assessed by the gamified assessment and the self-reported assessment showed a positive and significant correlation for Machiavellianism (*ρ* = 0.35, *p* < 0.001), narcissism (*ρ* = 0.23, *p* < 0.001), psychopathy (*ρ* = 0.19, *p* < 0.001), and sadism (*ρ* = 0.40, *p* < 0.001). In addition, the gamified assessment of dark personality was negatively and significantly related to H-H (*ρ* = −0.32, *p* < 0.001). The relationships of the gamified assessment of dark personality were negatively and significantly related to TP (*ρ* = −0.15, *p* < 0.05). The relationships of the CWBs presented a similar pattern in both assessments: in the self-reported assessment, the correlations for Machiavellianism were ρ_CWBO_ = 0.28, *p* < 0.001; *ρ*_CWBI_ = 0.21, *p* < 0.001; for narcissism, they were *ρ*_CWBO_ = 0.11, *p* < 0.05; *ρ*_CWBI_ = 0.12, *p* < 0.05; for psychopathy, they were *ρ*_CWBO_ = 0.36, *p* < 0.001; *ρ*_CWBI_ = 0.27, *p* < 0.001; and for sadism, they were *ρ*_CWBO_ = 0.37, *p* < 0.001; *ρ*_CWBI_ = 0.37, *p* < 0.001, while in the gamified assessment, the associations with dark personality were *ρ*_CWBO_ = 0.20, *p* < 0.001; and *ρ*_CWBI_ = 0.18, *p* < 0.01. Likewise, the dark personality assessed with the new gamified version of VASSIP was positively and significantly related to the scores obtained in propensity to moral disengagement (*ρ* = 0.30, *p* < 0.001) and, in turn, the latter was positively and significantly linked to CWBs (*ρ*_CWBO_ = 0.35, *p* < 0.001; *ρ*_CWBI_ = 0.29, *p* < 0.001).

**Table 3:** Correlations among demographic, personality, and workplace behavior variables.

Variables	1	2	3	4	5	6	7	8	9	10	11	12	13	14	15	16	17
1. Age	--																
2. Gender	−0.10* −0.21/−0.02	--															
3. Dark personality (VASSIP)	−0.20*** −0.29/−0.11	−0.28*** −0.37/−0.18	--														
4. SD4_Machiavellianism	−0.08 −0.17/0.01	−0.14** −0.23/−0.04	0.35*** 0.26/0.44	--													
5. SD4_Narcisism	0.02 −0.07/0.13	−0.07 −0.18/0.02	0.22*** 0.12/0.32	0.29*** 0.19/0.37	--												
6. SD4_Psychopathy	−0.01 −0.09/0.11	−0.15** −0.24/−0.04	0.19*** 0.09/0.29	0.27*** 0.18/0.37	0.42*** 0.34/0.51	--											
7. SD4_Sadism	−0.09* −0.18/0.01	−0.42** −0.50/−0.33	0.41*** 0.32/0.49	0.45*** 0.35/0.51	0.25*** 0.15/0.34	0.42*** 0.32/0.51	--										
8. Honesty-Humility	0.13** 0.04/0.23	0.05 −0.04/0.16	−0.32*** −41/−0.22	−0.51*** –0.58/−0.41	−0.35*** –0.44/−0.25	−0.34*** –0.45/−0.27	−0.40*** -0.48/−0.30	--									
9. PMD	−0.04 −0.13/0.05	−0.12* −0.20/−0.08	0.30*** 0.20/0.39	0.46*** 0.36/0.54	0.29*** 0.20/0.40	0.42*** 0.33/0.50	0.43*** 0.35/0.51	−0.47*** –0.57/−0.40	--								
10. Negative Emotionality	−0.24*** −0.32/−0.14	−0.20*** –0.31/−0.11	0.09 −0.01/0.19	0.15** 0.04/0.24	−0.07 −0.19/0.01	0.16** 0.06/0.25	0.05 −0.05/0.14	−0.14** −0.25/−0.04	0.12* 0.03/0.21	--							
11. Extraversion	0.16** 0.05/0.26	−0.02 −0.12/0.08	−0.14** −0.24/−0.04	−0.12* −0.23/−0.01	0.33*** 0.24/0.43	0.08 0.01/0.19	−0.12* −0.22/−0.03	−0.03 −0.14/0.06	−0.10* −0.20/−0.01	−0.41*** −0.49/−0.31	--						
12. Open-Mindedness	0.08 −0.02/0.17	0.04 −0.04/0.16	−0.14** −0.24/−0.04	−0.07 −0.17/0.04	0.22*** 0.12/0.32	0.07 −0.02/0.18	−0.07 −0.16/0.03	0.07 −0.04/0.16	−0.08 −0.18/0.02	−0.04 −0.13/0.06	0.26*** 0.17/0.36	--					
13. Agreeableness	0.12* 0.01/0.21	0.15** 0.04/0.25	−0.32*** -0.41/−0.22	−0.30*** -0.38/−0.19	−0.13* −0.22/−0.02	−0.33*** -0.41/−0.21	−0.42*** -0.49/−0.33	0.34*** 0.26/0.44	−0.35*** -0.43/−0.25	−0.32*** −0.40/−0.19	0.23*** 0.11/0.31	0.11* 0.01/0.21	--				
14. Conscientiousness	0.25*** 0.16/0.35	0.02 −0.08/0.11	−0.14** −0.23/−0.04	−0.08 −0.18/0.02	0.05 −0.02/0.16	−0.23*** -0.31/−0.12	−0.16** -0.24/−0.04	0.17*** −0.07/0.27	−0.15** −0.25/−0.05	−0.36*** −0.44/−0.25	0.34*** 0.23/0.42	0.03 −0.06/0.14	0.28*** 0.17/0.36	--			
15. TP	0.15** 0.07/0.26	0.10* 0.01/0.20	−0.15* −0.27/−0.06	−0.01 −0.12/0.09	0.02 −0.07/0.13	−0.19*** -0.30/−0.10	−0.09* −0.20/−0.01	0.11* 0.01/0.21	−0.19*** -0.28/0.08	−0.23*** −0.31/−0.12	0.29*** 0.18/0.37	0.12* 0.09/0.20	0.23*** 0.13/0.32	0.55*** 0.49/0.63	--		
16. CP	0.09 −0.11/19	0.09 −0.12/0.19	−0.05 −0.15/0.04	−0.05 −0.16/0.03	0.30*** 0.23/0.42	0.05 −0.05/0.15	−0.07 −0.20/0.01	0.04 −0.05/0.14	−10* −0.21/−0.01	−0.23*** −0.31/−0.13	0.43*** 0.32/0.50	0.27*** 0.16/0.33	0.21*** 0.10/0.30	0.32*** 0.22/0.41	0.45*** 0.35/0.54	--	
17. CWBO	−0.22 −0.32/−0.12	−0.05 −0.16/0.03	0.20*** 0.10/0.30	0.28*** 0.16/0.38	0.11* 0.01/0.22	0.36*** 0.27/0.45	0.37*** 0.27/0.46	−0.32*** -0.41/−0.21	0.35*** 0.23/0.43	0.25*** 0.14/0.34	−0.25*** −0.35/−0.15	−0.01 −0.12/0.09	−0.35*** −0.43/−0.24	−0.49*** −0.56/−0.41	−0.34*** −0.42/−0.24	−0.22*** -0.31/−0.12	
18. CWBI	−0.06 −0.04/0.15	−0.14** −0.24/−0.05	0.18*** 0.09/0.29	0.21*** 0.08/0.30	0.12* 0.03/0.23	0.27*** 0.18/0.37	0.37*** 0.26/0.45	−0.24*** -0.33/−0.13	0.29*** 0.18/0.37	0.14*** 0.04/0.24	0.00 −0.09/0.10	−0.09 −0.18/0.02	−0.32*** -0.41/−0.22	−0.18*** −0.28/−0.09	−0.19*** −0.28/−0.09	−0.03 −0.14/0.06	0.40*** 0.32/0.50

PMD: propensity to moral disengagement; TP = task performance; CP = contextual performance; CWBO: counterproductive work behaviors targeting the organization; CWBI: counterproductive work behaviors targeting individuals. 95% CI in columns with two values. *p < 0.05. **p < 0.01. ***p < 0.001.

To further explore the association between the brief measure that analyzes dark personality through a hard gamified assessment (GRA) and job performance, we developed four hierarchical regression models where dark personality was proposed as a predictor of dimensions of job performance (TP, CP, CWBO, and CWBI) as criteria. As can be seen in Table 4, the percentages of explained variance of the VASSIP measure of dark personality were significant for TP (1%) and both types of CWBs (3.1% for CWBO and 2% for CWBI).

**Table 4:** Hierarchical regression models predicting different aspects of job performance from dark personality and Big Five traits.

Dark personality (VASSIP)	TP					CP					CWBO					CWBI				
	*β*	SE	*t*	*p*	95% CI	*β*	SE	*t*	*p*	95% CI	*β*	SE	*t*	*p*	95% CI	*β*	SE	*t*	*p*	95% CI
Dark personality	−0.11	0.01	−2.24	0.03	[−0.05, −0.01]	−0.03	0.01	−0.50	0.68	[−0.03, 0.02]	0.18	0.16	3.61	< 0.001	[0.27, 0.91]	0.15	0.08	2.99	0.00	[0.09, 0.41]
Adjusted *R*^2^	0.01					−0.01					0.03					0.02				
*F*	5.01*					0.25					13.05***					8.92**				

Big Five and Dark personality	*β*	SE	*t*	*p*	95% CI	*β*	SE	*t*	*p*	95% CI	*β*	SE	*t*	*p*	95% CI	*β*	SE	*t*	*p*	95% CI
Dark personality	−0.02	0.01	−0.52	0.61	[−0.03, 0.02]	0.11	0.01	2.23	0.03	[0.01, 0.05]	0.06	0.15	1.27	0.20	[−0.10, 0.48]	0.02	0.08	0.45	0.65	[−0.12, 0.20]
Negative emotionality	−0.01	0.01	−0.27	0.79	[−0.02, 0.02]	0.01	0.01	0.27	0.79	[−0.01, 0.02]	0.01	0.10	0.12	0.90	[−0.18, 0.21]	0.08	0.05	1.41	0.16	[−0.03, 0.18]
Extraversion	0.05	0.01	0.94	0.35	[−0.01, 0.02]	0.29	0.01	5.43	<0.001	[0.03, 0.07]	−0.02	0.11	−0.43	0.67	[−0.27, 0.18]	0.19	0.06	3.40	<0.001	[0.09, 0.34]
Open-Mindedness	0.08	0.01	1.68	0.09	[−0.01, 0.03]	0.18	0.01	3.85	<0.001	[0.02, 0.05]	0.05	0.10	1.02	0.31	[−0.10, 0.30]	−0.07	0.06	−1.36	0.17	[−0.19, 0.03]
Agreeableness	0.01	0.01	0.22	0.82	[−0.02, 0.02]	0.14	0.01	2.72	0.01	[0.01, 0.05]	−0.24	0.13	−4.81	<0.001	[−0.91, −0.38]	−0.38	0.07	−7.22	<0.001	[−0.68, −0.39]
Conscientiousness	0.51	0.01	10.57	<0.001	[0.06, 0.09]	0.20	0.01	4.02	<0.001	[0.02, 0.05]	−0.41	0.10	−8.36	<0.001	[−1.04, −0.65]	−0.08	0.06	−1.48	0.14	[−0.19, 0.03]
Adjusted *R*^2^	0.30					0.25					0.29					0.17				
∆*R*^2^	0.30					0.26					0.27					0.16				
*F*	27.71***					22.01***					27.20***					14.40***				
*∆F*	31.85					26.34					29.06					15.16				

TP = task performance; CP = contextual performance; CWBO: counterproductive work behaviors targeting the organization; CWBI: counterproductive work behaviors targeting individuals.

Hierarchical regression analyses revealed that dark personality did not significantly predict job performance when Big Five traits were included in the models, with the exception of CP, where a lower dark personality score was positively associated with higher scores in this dimension of job performance.

## 4 Discussion

Video games have become an integral part of daily life, especially following the COVID-19 outbreak (Lewinson et al., 2024). Consequently, their influence is permeating other contexts, such as the assessment of individuals in organizational settings (Ramos-Villagrasa et al., 2022). At the same time, the interest in measuring dark personality is growing. Self-report assessments are limited by the risk of response manipulation (Wille et al., 2023), a difficulty that is especially relevant in the case of dark personality. Hence, in recent years, a notable effort has been made to propose alternative measures to self-reported questionnaires for personality assessment (Miller et al., 2019), such as GRAs (Ramos-Villagrasa and Naryniecki, 2025). The present study has proposed a hard gamified assessment to briefly measure dark personality in organizational contexts. The following sections discuss the study’s findings and their theoretical and practical implications.

First, it seems that the use of SJT items in serious games to measure dark personality is adequate. As Lievens and Motowidlo (2016) point out, SJTs allow a more accurate assessment of behaviors in specific situations by focusing on how individuals respond to work-relevant scenarios. Therefore, their situational approach can capture more accurately how candidates might display these characteristics in real work situations, providing added value in personnel selection and the study of dark personality in organizational settings. This is crucial in contexts where the concealment of dark personality traits may be particularly detrimental, such as in selection processes where impression management is more likely among individuals with these traits (e.g., Curtis et al., 2022).

Regarding content validity, the indices of the VASSIP extension are considered “adequate or good” because they reach Kendall’s *W* above 0.60 (Cicchetti, 1994). This result suggests that the scenarios selected for the situational judgment test are suitable for measuring dark personality. To capture the underlying nature of the dark personality, a CFA was conducted to assess whether the generated scenarios loaded on a single factor, representing the so-called D factor, a construct resulting from the shared malevolence of several related dark traits (Moshagen et al., 2020). Initial results with this hard gamified assessment did not show adequate fit indices for a unidimensional model of dark personality. However, a five-item solution with satisfactory fit indices was achieved after eliminating items with low contributions to the model. This result suggests the existence of a set of maladaptive traits that would form a brief and coherent measure of dark personality (Rauthmann and Kolar, 2012). Therefore, the new extended version of VASSIP presents a novel solution for exploring dark personality in organizations that rely on personality assessments for HR processes. It is also important to acknowledge the practical implications of employing an instrument based on a unidimensional factor structure. Although the unidimensional model offers a theoretically parsimonious approach to capturing the shared tendency of dark personality to maximize personal gain at the expense of others, it does not account for the unique variance of each trait in predicting specific behavioral outcomes (Book et al., 2016). In this regard, the unidimensional configuration can obscure the qualitative distinctions among the various dark traits (Vize et al., 2020). Consequently, this instrument can provide a parsimonious and useful tool for the initial assessment of dark personality in personnel selection. However, it should be supplemented with additional evaluations when making decisions about targeted interventions for specific behavioral risks. Regarding convergent and concurrent validity, both were successfully tested. Correlational analyses have shown a moderate and significant association between the VASSIP measure of dark personality and the SD4 scale, indicating a moderate correspondence between the two dark personality assessment instruments. These findings suggest that, although the measures assess similar aspects of dark personality, they are not entirely equivalent (Rauthmann and Kolar, 2012). This pattern is consistent with previous research showing only modest convergence between situational judgment measures and traditional self-report inventories of personality (e.g., Arthur and Villado, 2008; Lievens and Motowidlo, 2016). Moreover, the results indicate that the SJT captures behavioral manifestations of dark personality, supporting its convergent validity. In addition, the SJT showed theoretically coherent relationships with external variables, such as lower honesty and integrity, higher moral disengagement, and greater tendencies toward counterproductive work behaviors, while its relationship with contextual performance was negligible. This configuration aligns with the dark core of personality framework (Moshagen et al., 2018), indicating that individuals displaying stronger dark tendencies in the SJT also exhibit patterns of behaviors consistent with this theoretical model. However, these predictive relationships are modest and should be interpreted with appropriate prudence, such magnitudes are typical and meaningful in personality-behavior research (Fernández-del-Río et al., 2020; Funder and Ozer, 2019). Therefore, even with small effects, the gamified SJT provides practically useful and ecologically valid insights that complement traditional self-report personality assessments.

Another interesting result is related to the regression analyses. Using the measure developed in this study as the sole predictor, it can predict TP and CWBs. Although the variance explained by this model is small, it indicates that this solution is appropriate for screening the associations between dark personality and these criterion variables. However, when the remaining personality variables measured in VASSIP are included, the effect of the dark personality measure becomes negligible. There are two complementary explanations for this result: (1) the predictive power of the Big Five for performance is greater than that of dark personality; thus, when using a brief measure of the latter construct, its effect diminishes; (2) Ramos-Villagrasa et al. (2022) suggest that GRAs with measures that are more like conventional tests (such as the Big Five measure in this study) perform better than those that are more “game-like” (such as the dark personality measure).

The observed validity coefficients of the VASSIP gamified assessment are comparable to those typically reported for traditional dark personality measures. Specifically, the correlations with CWBs and TP (*ρ* = −0.15) fall within the range found in meta-analyses of self-reported dark traits (O’Boyle et al., 2012) and in previous primary studies (Fernández-del-Río et al., 2020). These results suggest that, despite its brief and interactive format, the gamified SJT captures the core variance of the dark personality with a similar predictive strength to traditional self-report scales. However, hierarchical regression analyses indicated modest practical predictive value: the gamified measure explained a small proportion of variance in job performance and CWBs (Δ*R*^2^ = 0.01–0.03), and its contribution diminished when the Big Five traits were included. This pattern aligns with prior evidence that the predictive power of dark traits is often limited after controlling for broader personality factors (Moscoso and Salgado, 2004). Nevertheless, the practical advantage of the VASSIP extension lies in its reduced susceptibility to faking and its capacity to elicit authentic behavioral responses within work-relevant contexts (Landers and Collmus, 2022; Melchers and Basch, 2022). Hence, while the predictive magnitude is modest, the measure offers an ecologically valid and ethically sound alternative to traditional questionnaires for assessing dark personality in organizational settings.

Additionally, hierarchical regression analyses have shown that the gamified version of dark personality is a positive predictor of CP when considering the VASSIP measure of bright personality. From our point of view, this result could be due to a statistical artifact, as multicollinearity seems the most plausible explanation, given that these variables are related to personality (Daoud, 2017; Shrestha, 2020). This is further supported by the fact that when dark personality is analyzed in isolation, the same prediction did not occur. Nevertheless, this finding could also align with recent research suggesting that certain dark personality profiles may promote behaviors beneficial to the organization, if they result in personal gain (Kızıloğlu et al., 2021).

Finally, it is worth noting that the assessment of dark personality through the serious game VASSIP revealed significant differences according to the application condition, where applying the GRA first resulted in higher scores on the dark personality assessed through it. This result suggests, like prior literature, that gamified tests may be more resistant to faking. However, this requires further support from research.

Taking into account all the aforementioned, we can conclude that the new version of VASSIP has as main advantages: (1) the integration in the same GRA of the assessment of bright personality traits through the Big Five with a measure of dark personality; (2) their brief nature in terms of content (6 items to measure each of the Big Five traits and 5 items to measure dark personality), and application time (around 15 min); and, (3) according to the results of its previous version (Ramos-Villagrasa et al., 2024), the GRA is rated favorably by the people evaluated. All this leads us to conclude that VASSIP can be a useful instrument in contexts in which dark personality assessment is desired but is not considered necessary, or there is insufficient time to carry out in-depth evaluations. However, a more exhaustive analysis of the different existing dark profiles would require the use of more detailed and comprehensive scales.

From a general perspective, the present study supports the idea that GRAs may be an interesting way to measure dark personality in the workplace. However, the modest results suggest clearly that more research is needed to know how to develop better assessments based on games (Ramos-Villagrasa and Fernández-del-Río, 2023). Therefore, the use of gamified assessments in personnel selection context presents both promising opportunities and important ethical considerations. From a practical standpoint, these gamified-related assessment tools can capture behavioral tendencies that complement self-report measures, providing a richer understanding of candidates’ traits and potential workplace behaviors. Importantly, their implementation should support diversity and fairness in selection processes. The literature emphasizes that a diverse workforce offers substantial organizational benefits, which begin by ensuring that assessment tools provide equal opportunities to all candidates, regardless of gender, age, nationality, or other characteristics (Langer et al., 2023). Early research on gamified-related assessment tools suggests minimal bias, particularly regarding gender and educational background, though further work is needed to explore other diversity dimensions such as age and culture (Brown et al., 2022; Melchers and Basch, 2022; Ramos-Villagrasa et al., 2024).

Ethical considerations are equally critical. Although gamified assessments are designed to be valid and engaging, their excessive or improper use can unintentionally stress or influence participants in counterproductive ways. Kim and Werbach (2016) suggest that excessive use of gamified assessments can potentially infringe upon autonomy or negatively impact candidates’ well-being. Transparency about the purpose of the assessment, clear instructions, voluntary participation, and accessibility for all candidates are key to mitigating such risks. Furthermore, cultural factors should be considered when applying gamified tools in multinational contexts to avoid misunderstandings or inadvertent disadvantages. Overall, when carefully implemented, gamified assessments are ethically responsible, practically useful, and supportive of organizational diversity goals, while offering an engaging alternative to traditional assessment methods.

### 4.1 Limitations and further research

Like any study, the present one has some limitations that should be acknowledged. First, this study relies solely on self-report measures collected concurrently, which makes it susceptible to common method variance. Future research is crucial to address this, not only by utilizing multiple measurement time points and assessing job performance through supervisor ratings, but also by more thoroughly exploring the impact of common method variance in GRAs more broadly. Specifically, when evaluating GRAs’ incremental validity over self-report measures, it is vital to investigate how assessment methodology interacts with outcome measurement. As Barends et al. (2022) demonstrated, a game-based assessment of H-H showed incremental validity only for behavioral tasks, not for self-reported outcomes. Therefore, subsequent studies should incorporate multi-source or objective outcome measures to provide a more robust evaluation of GRAs’ unique predictive contributions, moving beyond solely self-reported criteria. Second, our GRA is a hard-gamified assessment tool. Previous research suggests that soft-gamified assessment tools obtain better results in terms of validity, but we did not compare soft and hard versions (e.g., introducing the SD4 items in the GRA vs. the current version of VASSIP). However, we believe that soft gamified assessment is not an adequate option for measuring dark personality, as it is more challenging to develop a shadow assessment, and applicants’ reactions are less favorable. Third, our study focused on developing a brief measure of dark personality but did not verify whether this version is less prone to faking. This should be verified in further studies. Fourth, the study employs a cross-sectional design, which precludes the establishment of causal relationships between dark personality and the outcomes measured. While the current design allows for the assessment of associations and initial validation of the gamified-related assessment, longitudinal studies are required to examine causal links and changes over time. Fifth, the study lacks longitudinal predictive data. Consequently, while the current findings provide evidence of the VASSIP’s reliability and construct validity, its predictive utility for real-world outcomes or future behaviors remains to be established. Future research should incorporate longitudinal designs to assess the stability of measured traits and the instrument’s ability to predict relevant behavioral outcomes over time. Sixth, another relevant limitation is the capitalization on chance associated with the item selection used to construct VASSIP’s dark personality measure. This likely overestimates the strength of the relationships analyzed. Consequently, a replication study is required to more robustly ascertain the true relationship between VASSIP’s dark personality measure and the remaining variables.

Continuing with further research, applicant reactions to the present version of VASSIP should be gathered. In Likert-type scales, items directly associated with dark personality promote negative reactions, but we do not know if this is true in the SJT-gamified version. This could interest researchers and designers of measurement instruments of socially undesirable constructs. In this regard, although the present study focused on the psychometric evaluation of an extended version of the VASSIP for initially assessing dark personality in personnel selection contexts, future research should consider testing a multidimensional alternative model, such as a higher-order or bifactor structure. Such an approach would allow the examination of both the shared variance captured by the D factor and the unique contributions of individual dark personality traits, potentially enhancing predictive precision and providing a more nuanced understanding of behavioral outcomes in organizational settings. Additionally, we consider it necessary to compare different types of GRAs that measure personality to verify possible differences depending on the degree of playfulness.

## Data Availability

The datasets presented in this study can be found in online repositories. The names of the repository/repositories and accession number(s) can be found below: Open Science Framework (OSF): https://osf.io/vdfgw/?view_only=dbb2afb1c85c460e906ed32297a648bc.
