# Supplementary material for: Using a serious game for a brief assessment of dark personality in the workplace
Source: Front Psychol. 2026 Jan 26;16:1686784. doi: 10.3389/fpsyg.2025.1686784 (PMC12883365; doi:10.3389/fpsyg.2025.1686784)
Supplement: Supplementary file 1 [file Table_1.DOCX]

Items of the Dark Personality Gamified Measure

# Introduction

It is necessary to note that, as is typical of hard gamified assessments (Ramos-Villagrasa & Naryniecki, 2025), the items may not be fully understood as they are presented without the preceding narrative. Values in brackets [] indicate the score assigned to each response alternative.

# Items

Item 5

*You continue walking down the hallway until you find a functional terminal. It's a chatbot that you usually use to report incidents. Without hesitation, you type 'Help' to activate it. Instead of greeting you as usual, the screen returns a question for you:*

*'Why do you deserve help?'*

- You type 'I am a person, and all people deserve help.' [1]

- You type 'This space station needs someone with my talent.' [3]

- You type 'I am more capable than the rest of the crew on this ship.' [5]

Item 7

*It opens, revealing a long corridor ahead of you. At the other end is a worker you don't recognize. As soon as she sees you, the worker presses the button to close the door next to her and runs away from you, as if she were afraid of you. The woman runs so fast that she trips over a cable and falls flat on her face. You see her writhe in pain before the door closes.*

- You shrug... you would have liked to talk to her. [1]

- You smile... she deserved it for being so reckless. [3]

- You laugh... it's funny when stupid people act foolish." [5]

Item 8

*You walk down one of the hallways and notice that the door to the small human resources office is open. You take a look inside for anything useful, but you only find the report of the ten highest-rated personnel for VASSIP-2.*

- You check to see if you are one of the people included in the list. [1]

- You take a look to see where on the list you are. [3]

- You don't need to look at the list: you know your name tops it. [5]

Item 10

*You know you are safe here because the room is perfectly soundproofed to prevent any conversation from being heard outside. You can allow yourself to release all the tension that has built up since you woke up in your room.*

- You release the tension by stretching with the help of the table. [1]

- You release the tension by kicking the chairs. [3]

- You release the tension by hitting the computer until it shatters. [5]

Item 11

*You are still observing everything when you trip over a glass jar that breaks into many pieces. A small fire starts. The situation could become dangerous very quickly.*

- You leave the laboratory as quickly as possible to avoid the fire. [1]

- You quickly examine the part of the laboratory that has not caught fire. [3]

- You take your time to examine the entire laboratory despite the fire. [5]
